# A rare presentation of dens in dente in the mandibular third molar with extra oral sinus

**DOI:** 10.4103/0973-029X.72508

**Published:** 2010

**Authors:** Monika Bansal, NN Singh, Anand Pratap Singh

**Affiliations:** *Faculty of Dental Sciences, Institute of Medical Sciences, Banaras Hindu University, Varanasi, India*; 1 *Department of Oral Pathology and Oral Microbiology, Kothiwal Dental College and Research Centre, Moradabad, India*; 2 *Department of Oral Medicine & Radiology, Kothiwal Dental College and Research Centre, Moradabad, India*

**Keywords:** Dense in dente, extra oral sinus, mandibular molars

## Abstract

The unusual case of dense in dente in mandibular molar area with extra oral sinus in a 30-year-old female is presented. The chief complaint of the patient was wound formation and pus discharge from the right side of lower jaw for many years. Clinical examination revealed extra oral sinus and mild swelling in vestibular region opposite the right mandibular molars. On radiographical examination, right mandibular third molar had bulbous root associated with periapical radiolucency. It appeared that there was a tooth within a tooth and the invagination extended nearly to the root apex. A clinical diagnosis of dense in dente Type III was confirmed by stereomicroscopy of ground section of the tooth. Extra oral sinus healed after extraction of the mandibular third molar within a month.

## INTRODUCTION

Dens in dente, also known as dens invaginatus or dilated composite odontome is a rare developmental tooth anomly characterized by invagination of the enamel organ into the dental papilla. The frequency of dens in dente is more common in permanent maxillary lateral Incisors[1]. In the madibular area, this anamoly is rarely present. Thus, we are presenting a type III dens in dente in the right mandibular third molar region, rare location for occurence.

## CASE REPORT

**Figure 1:**
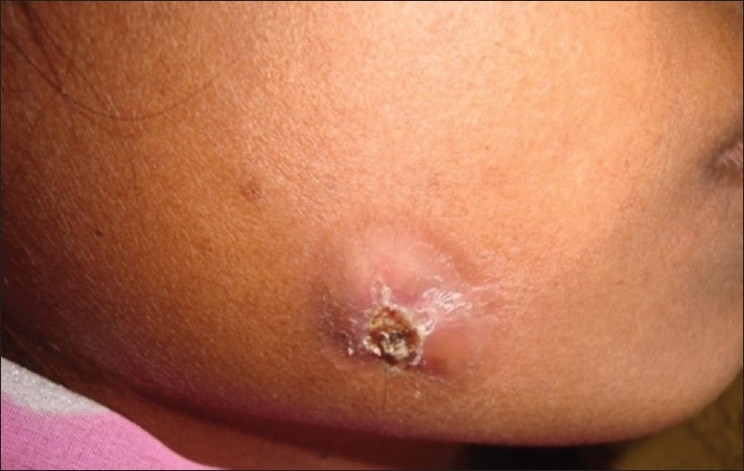
Sinus with pus discharge on the facial side in the right mandibular molar area

**Figure 2:**
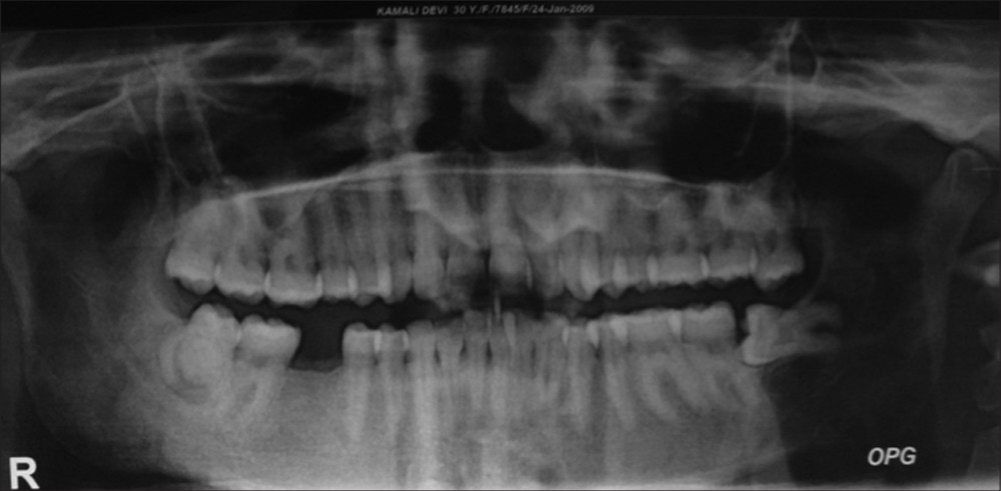
OPG showing dense in dente in right mandibular third molar

**Figure 3:**
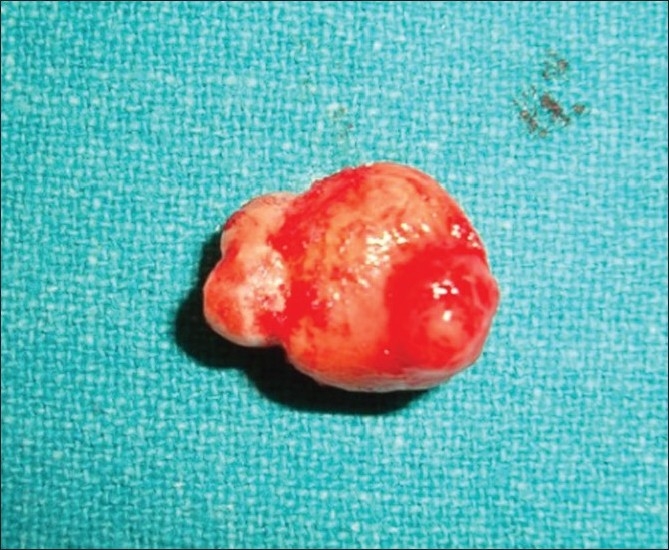
Extracted mandibular third molar with a very bulbous root and periapical pathology

**Figure 4:**
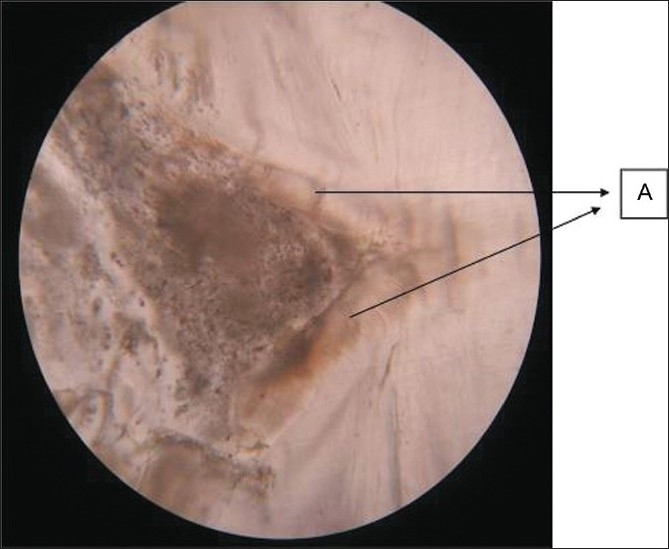
Ground section of pulp chamber at high magnification (40×) showing “dense in dente” (A-enamel layer)

**Figure 5:**
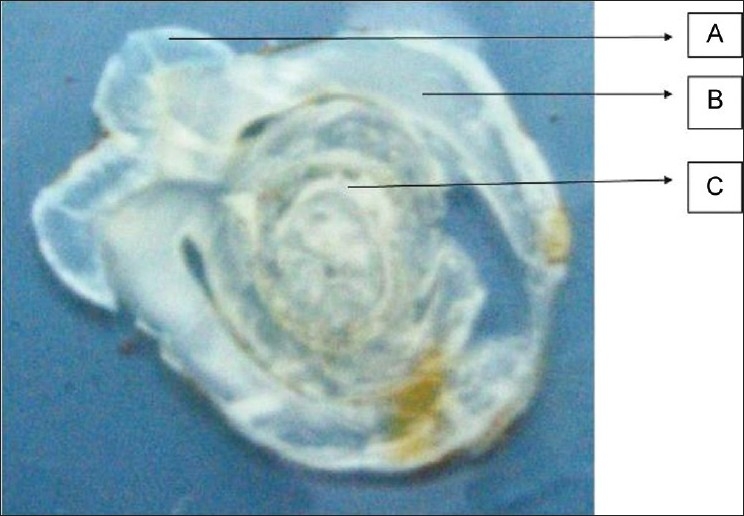
Stereomicroscopic view of ground section represents “dense in dente” (A- enamel, B- dentin, C- pulp chamber showing enamel, dentin, pulp)

**Figure 6:**
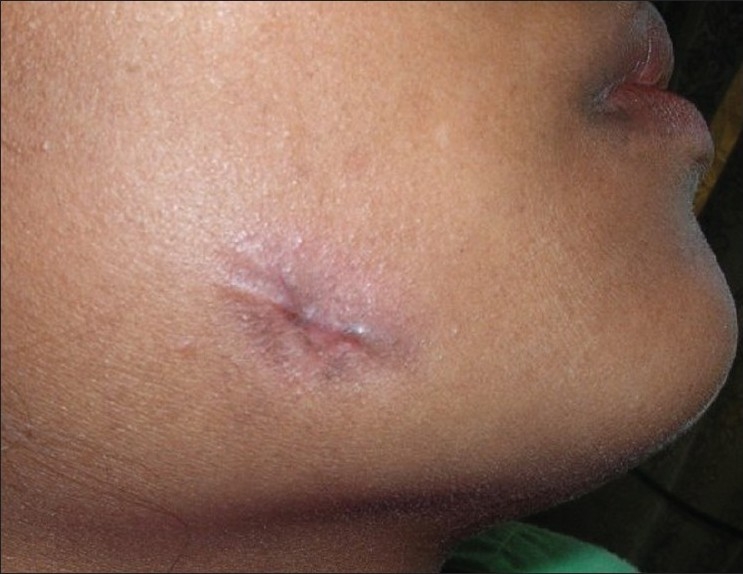
Healed extra oral sinus

A 30-year-old female patient reported in dental OPD of Sir Sunder Lal Hospital, Faculty of Dental Sciences, Institute of Medical Sciences, Banaras Hindu University, Varanasi with a chief complaint of wound formation and pus discharge from the right side of lower jaw for many years. The general health of the patient was normal and her medical history revealed no significant problem. Extraoral examination revealed facial sinus with pus discharge from the body of the mandible [Figure 1]. On intraoral examination, there was mild swelling in the vestibular region opposite the mandibular molars. No other significant changes were present. On radiographical examination, the right mandibular third molar had the bulbous root associated with periapical radiolucent area. It became evident that there was a tooth within a tooth with invagination extending nearly to the root apex [Figure 2]. A clinical diagnosis of dens in dente Type III was made. At that time, the extraction of mandibular third molar was planned as it was believed that extra oral sinus may be due to that tooth. The extracted tooth had a very bulbous root along with periapical pathology [Figure 3]. Stereomicroscopy of ground section of tooth confirmed the diagnosis by showing enamel, dentin and pulp within a pulp chamber [Figures 4, 5]. The extra oral sinus healed within a month following extraction of mandibular third molar [Figure 6].

## DISCUSSION

Dens in dente is a rare developmental tooth anomaly characterized by invagination of the enamel organ into the dental papilla that begins at the crown and often extends to the root even before the calcification of the dental tissues. Dens in dente is more common in permanent maxillary lateral incisors (0.25-5.1%),[1] but rare in molars. Dens in dente is classified by Oehlers[2] into three types, depending upon its extent into the crown, root and root apex. The mild form is more common than severe one. Various treatment methods have been reported including conservative restorative treatment, nonsurgical root canal treatment and surgical treatment like endodontic surgery, intentional replantation, and extraction.[3] Generally, Type l and Type ll do not present any problem during the endodontic treatment because invagination does not reach upto the apical region of canal and restricts to the interior of the canal. However, in Type lll, nonsurgical endodontic treatment is difficult because invagination may reach the root apex of the tooth. Even if, Type lll is treated in anterior teeth through conventional endodontic treatment in the area of invagination supplemented with retrograde filling of the foramen of the principal canal in some cases, the retrograde filling is difficult in posterior teeth.[4, 5] In this case report, the mandibular third molar presented with periapical radiolucency that might have been the cause of extra oral sinus due to infected root canals. Surgical endodontic therapy in mandibular third molars is difficult because of their position. In addition, invaginated teeth present technical difficulties with respect to the management of the complicated root canal morphology. Therefore, this tooth was extracted and the extra oral sinus healed within a month. Thus, it is concluded that dens in dente is an important anomaly that may lead to pulp infection and periapical pathosis through invagination. Thus, an early detection and sealing of the openings with restorative material is necessary to prevent further complications.
